# Mitochondrial haplogroups in patients with rheumatoid arthritis: No association with disease and disease manifestations

**DOI:** 10.1371/journal.pone.0188492

**Published:** 2017-12-20

**Authors:** Pernille Hurup Duhn, Jacob Sode, Christian Munch Hagen, Michael Christiansen, Henning Locht

**Affiliations:** 1 Department of Rheumatology Frederiksberg Hospital, Nordre Fasanvej, Frederiksberg, Denmark; 2 Department of Autoimmunology and Biomarkers, Statens Serum Institut, Artillerivej, Copenhagen S, Denmark; 3 Institute of Regional Health Research, Center Sønderjylland, University of Southern Denmark, Campusvej, Odense M, Denmark; 4 Department for Congenital Disorders, Statens Serum Institut, Artillerivej, Copenhagen S, Denmark; 5 Department of Biomedical Sciences, Faculty of Health and Life Science, University of Copenhagen, Blegdamsvej, Copenhagen N, Denmark; Universidade do Porto Instituto de Patologia e Imunologia Molecular, PORTUGAL

## Abstract

**Objective:**

To describe the distribution of specific mitochondrial DNA (mtDNA) haplogroups (hgs) in a cohort of patients with rheumatoid arthritis (RA).

**Methods:**

Two-hundred nineteen consecutive patients with RA had mtDNA isolated from their blood, sequenced and haplotyped. Patients were diagnosed according to the American College of Rheumatology (ACR)/European league against Rheumatism (EULAR) criteria. Demographic and clinical data were retrieved from the Danish nationwide database (DANBIO). Logistic regression analyses were performed to test for associations.

**Results:**

One-hundred eighty-four patients were eligible for analysis. Haplogroup frequencies were: H (n = 88; 47.8%), U (n = 37; 20.1%), T (n = 22; 12.0%), J (n = 16; 8.7%), K (n = 11; 5.9%), HV (n = 6; 3.3%) and V (n = 4; 2.2%). The distribution of individual hgs was identical to the background population. Radiographic erosions were significantly associated with hg clusters JT (OR = 2.37, 95% confidence interval (CI): 1.07–5.53, p = 0.038). Significantly fewer patients from hg cluster JT received biological treatment (OR = 0.17, 95% CI: 0.03–0.87, p = 0.038). Albeit, none of these associations were significant when corrected for multiple tests.

**Conclusion:**

There was no significant association between mtDNA hgs and presence of RA or disease manifestations. There was an, albeit insignificant, overrepresentation of patients with hg JT among patients with erosive disease; however, slightly fewer patients in the JT group were treated with biological drugs.

## 1. Introduction

Mitochondrial DNA (mtDNA) has a strict maternal mode of inheritance and is not subject to recombination [1]. Every mitochondrion contains from 2–5 copies of mtDNA [1]. Throughout evolution mutations have accumulated sequentially in the 16.6 kb mtDNA genome leading to the fixation of haplogroups (hgs) [1,2]. Individual hgs are defined by the combination of single nucleotide polymorphisms (SNP) and are named from hg A to hg Z [1,3].

mtDNA encodes for proteins involved in the mitochondrial production of adenosine triphospate (ATP) by oxidative phosphorylation (OXPHOS), a process also involved in generation of superoxide and other reactive oxygen species (ROS) [1–3]. Furthermore, the mitochondria are of importance for apoptosis, calcium handling and several intracellular signalling pathways [4,5]. Mutations in mtDNA have been recognised as causes of disease often involving organs with high energy turnover [4]. This has resulted in the formulation of a bio-energetic paradigm suggesting that variation in mtDNA may lead to changes in oxidative stress in cells and eventually to differences in morbidity, mortality, and longevity [4–6].

Several studies have investigated the association between hgs and various diseases [1,2,7–12]. Rego-Perez et al. found that mtDNA haplogroup (hg) J lowered the risk for osteoarthritis (OA) in the knees and hips [10] and others have found that hgs J and K are associated with a reduced risk of Parkinson’s disease [8]. Furthermore, hg U has been linked with Alzheimer’s disease [9], and hgs H and HV have been associated with the development of hypertrophic cardiomyopathy [1]. Recently, a large study identified several mtDNA SNPs each associated with several degenerative diseases such as Parkinson’s disease, multiple sclerosis and ischaemic stroke [13].

Rheumatoid arthritis (RA) is a chronic auto-immune disorder which is characterized by synovial inflammation and hyperplasia, autoantibody production, cartilage and bone destruction leading to erosions and joint deformity [14]. A recent study has suggested ROS to be involved in the pathogenesis of RA [15], but whether this relates to associations between RA and the mtDNA hgs have not previously been investigated. The aim of this study was to analyse the distribution of mtDNA hgs in a cohort of patients with RA according to disease phenotype, presence of rheumatoid factor, and biological treatment. The distribution of mtDNA hgs in the RA-cohort was compared with two historical control groups from the background population.

## 2. Materials and methods

### 2.1. Study population and data collection

Consecutive randomly selected patients treated for RA in the out-patient clinic at the Department of Rheumatology, Frederiksberg Hospital, Copenhagen, Denmark between January-December 2013 were assessed for inclusion. Inclusion criteria were fulfilment of the classification criteria for RA proposed by the American College of Rheumatology (ACR) in 1987 and European league against Rheumatism (EULAR) in 2010 [16], age above 18 years and written informed consent to have blood analysed for mtDNA haplotyping and to have data extracted from relevant databases. Exclusion criterion was a non-European hg e.g. only patients with hg H, hg HV, hg V, hg U, hg K, hg J or hg T were included [17]. This exclusion was performed to avoid that the association analysis should be skewed due to population stratification.

Demographic and clinical data were extracted from the nationwide DANBIO database (The National Danish Registry for Biological Treatment of Rheumatic Diseases), which contains prospective data on all adult Danish rheumatologic patients subjected to both biological treatment and conventional treatment with synthetic disease modifying anti-rheumatic drugs (sDMARD). Data included gender, age, IgM rheumatoid factor (IgM-RF) and anti-cyclic citrullinated peptide (anti-CCP) status, radiographic joint erosions and whether the patients were receiving biological/sDMARDs treatment. Furthermore, the disease activity score 28-joints with c-reactive protein (DAS28-CRP) was registered. By convention DAS28-CRP levels can be graded in four categories according to the disease score: “High disease activity” (> 5.1), “Moderate disease activity” (3.2 ≤ 5.1), “Low disease activity” (2.6 ≤ 3.2) and “Remission” (< 2.6) [18]. Biological treatment is considered when the patient has current and persistent moderate or high disease activity [19]. Therefore, when testing the associations between clinico-pathological factors and allocation to biological treatment the DAS28-CRP was divided into”remission/mild” or “moderat/severe” disease activity. Biological treatment may also be considered if x-ray show current, progressive erosions regardless of the DAS28-CRP [19].

The distribution of the mtDNA hgs in the study population were compared with results from two other Danish studies performed in the Greater Copenhagen area, Denmark. The study by Benn et al. examined mtDNA hg distribution in 9254 patients from the Copenhagen City Heart Study [6]. The other study by Mikkelsen et al. analysed the mtDNA hg distribution from 206 employees at the Department of Forensic Medicine, Faculty of Health Sciences, University of Copenhagen and the Danish Police force [20].

### 2.2 mtDNA haplotyping

DNA was extracted from the blood using the Maxwell® 16 system (Promega Corporation, Madison, Wisconsin, USA). Polymerase Chain Reaction (PCR) and pyrosequencing primers for mtDNA haplotyping were designed using PyroMark Assay Design 2.0 and the revised Cambridge Reference Sequence (rCRS) (NC_012920) as template. The combination of diagnostic SNPs used for mtDNA haplotyping of European mitochondrial hg H, hg U, hg K, hg J, hg T, and hg V is presented in Table 1. The hgs were classified according to PhyloTree.org (mtDNA tree Build 16 (19^th^ of February 2014)) [1,17].

**Table 1:** European mitochondrial haplogroups [1–2,17].

rCRS position	Diagnostic SNP for haplogroup (variant)	
7028	H (C);	U, K, J, T, V, HV (T)
11467	K, U (G);	H, J, T, V, HV (A)
10550	K (G);	H, U, J, T, HV, V (A)
15607	T (G);	H, U, K, J, HV, V (A)
16069	J (T);	H, U, K, T, HV, V (C)
11251	J, T (G);	H, U, K, HV, V (A)
4580	V (A);	H, U, K, J, T, HV (G)
14766	HV, V (C);	H, U, K, J, T (T)

rCRS: revised Cambridge Reference Sequence; SNP: single nucleotide polymorphism.

### 2.3 Statistical analysis

Due to small sample sizes we pooled the hgs into evolutionary closely related hg clusters: HV, UK and JT.

Logistic regression analyses were performed to test for associations between the hg clusters and anti-CCP (negative/positive), IgM-RF (negative/positive), DAS28-CRP (remission/mild or moderate/severe), erosions (absent/present) and biological treatment (no/yes). Multivariable analyses were performed of associations between clinico-pathological factors and allocation to biological treatment. A two-tailed p-value of 0.05 was used as level of significance. The results are presented in odds ratio (OR). Statistical analyses were performed in R version 2.15.2 [21].

### 2.4 Ethics statement

The Regional Ethics Committee of Copenhagen (M-20100153) and the Danish Data Protection Agency (J. 2010-41-4719) approved this study. The study was conducted in accordance with the Declaration of Helsinki. Patients did not give informed consent as the samples were taken as part of routine medical care. This in accordance with the permission from the Regional Ethics Committee of Copenhagen and The Declaration of Helsinki, para 32. The authors were not involved in drawing blood and collecting of samples, but the samples were blinded prior to laboratory analyses.

## 3. Results

In total, 219 patients were included. They were diagnosed with seropositive RA (n = 185), seronegative RA (n = 33), or juvenile arthritis (n = 1). Thirty-five patients were excluded from analysis due to having a non-European hg (n = 29) or an unknown hg (n = 6). The distribution of mtDNA hgs and hg clusters was analysed in all included patients.

Patient demographics and clinical data stratified by the hgs and hg clusters are presented in Table 2. Median age was 60.5 years (interquartile range 22–88 years).

**Table 2:** Patient characteristics according to haplogroups and haplogroup clusters.

	Total	HV	H	V	Cluster HV	U	K	Cluster UK	T	J	Cluster TJ
	184	6	88	4	98	37	11	48	22	16	38
**Gender:**											
Female	137	5	61	3	69	28	10	38	19	11	30
Male	47	1	27	1	29	9	1	10	3	5	8
**Age groups (years):**											
≤40	22	2	12	0	14	2	1	3	2	3	5
>40–50	24	2	9	0	11	7	1	8	3	2	5
>50–60	46	2	22	3	27	6	5	11	8	0	8
>60–70	49	0	25	0	25	12	2	14	3	7	10
>70–80	36	0	19	1	20	6	1	7	5	4	9
>80	7	0	1	0	1	4	1	5	1	0	1
**Anti-CCP:**											
ND^#^	45	0	24	0	24	10	3	13	3	5	8
Positive	108	5	51	3	59	20	7	27	12	10	22
Negative	31	1	13	1	15	7	1	8	7	1	8
**IgM-RF:**											
ND^#^	16	0	6	0	6	5	2	7	2	1	3
Positive	146	4	76	3	83	26	9	35	14	14	28
Negative	22	2	6	1	9	6	0	6	6	1	7
**DAS28-CRP:**											
ND^#^	1	0	1	0	1	0	0	0	0	0	0
Remission (<2.6)	34	0	16	1	17	10	2	12	2	3	5
Mild (2.6≤3.2)	13	0	8	0	8	1	1	2	0	3	3
Moderate (3.2≤5.1)	74	3	31	2	36	17	4	21	11	6	17
Severe (>5.1)	62	3	32	1	36	9	4	13	9	4	13
**Erosions:**											
ND^#^	10	0	3	1	4	3	1	4	2	0	2
Present	97	1	43	2	46	19	7	26	14	11	25
Absent	77	5	42	1	48	15	3	18	6	5	11
**Biological treatment:**											
ND^#^	1	0	1	0	1	0	0	0	0	0	0
Yes	131	6	65	4	75	23	8	31	17	8	25
No	52	0	22	0	22	14	3	17	5	8	13

# Not defined/No registered data.

Anti-CCP: anti-cyclic citrullinated peptide; IgM-RF: IgM rheumatoid factor; DAS28-CRP: disease activity score 28-joints with c-reactive protein; Erosions: radiographic joint erosions.

The hg frequencies are shown in Table 3. Furthermore, hg frequencies from two control groups from the background population are listed in Table 3 [6,20]. In the study by Benn et al. no data on the hg HV [6] were available. Mikkelsen et al. had no data on hg V [20]. The distribution of individual hgs was comparable with the distribution of the background populations.

**Table 3:** The frequencies and distribution of mtDNA haplogroups in the rheumatoid arthritis patient cohort and two control cohorts from the Danish background population.

	RA cohort n = 184 (%)		Benn et al. n = 8451 (%)		Mikkelsen et al. n = 177 (%)	
**Haplogr.cluster HV**						
hg HV	6	(3.3)	-	-	7	(4.0)
hg H	88	(47.8)	4244	(50.2)	82	(46.3)
hg V	4	(2.2)	412	(4.9)	-	-
**Total**	**98**	**(53.3)**	**4656**	**(55.1)**	**89**	**(50.3)**
**Haplogr.cluster UK**						
hg U	37	(20.1)	1469	(17.4)	26	(14.7)
hg K	11	(5.9)	571	(6.8)	20	(11.3)
**Total**	**48**	**(26.0)**	**2040**	**(24.2)**	**46**	**(26.0)**
**Haplogr.cluster JT**						
hg T	22	(12.0)	912	(10.8)	17	(9.6)
hg J	16	(8.7)	843	(9.9)	25	(14.1)
**Total**	**38**	**(20.7)**	**1755**	**(20.7)**	**42**	**(23.7)**

RA: rheumatoid arthritis; Benn et al.: Benn M, Schwartz M, Nordestgaard BG, Tybjærg-Hansen A. Mitochondrial haplogroups: Ischemic cardiovascular disease, other diseases, mortality, and longevity in the general population. Circulation. 2008; 117: 2492–2501. 10.1161/CIRCULATIONAHA.107.756809 18458168. [6]; Mikkelsen et al.: Mikkelsen M, Sørensen E, Rasmussen EM, Morling N. Mitochondrial DNA HV1 and HV2 variation in Danes. Forensic Sci Int Genet. 2010; 4: 87–88. 10.1016/j.fsigen.2009.07.007 20457038. [20].

A significant association between hg clusters and disease activity was found with regard to erosions as patients with hgs in cluster JT showed a significantly higher frequency of erosions (OR = 2.37, 95% CI: 1.07–5.53, p = 0.038) compared to hg cluster HV, Table 4.

**Table 4:** Associations between haplogroup clusters and univariable clinicopathological data.

	Erosions			DAS28-CRP			Anti-CCP			IgM-RF			Bio.treatment		
	OR	(95% CI)	*P*	OR	(95% CI)	*P*	OR	(95% CI)	*P*	OR	(95% CI)	*P*	OR	(95% CI)	*P*
**HV**	1.00			1.00			1.00			1.00			1.00		
**UK**	1.51	0.73–3.14	0.267	0.84	0.39–1.85	0.665	0.86	0.33–2.35	0.757	0.88	0.29–2.99	0.823	0.53	0.25–1.15	0.106
**JT**	2.37	1.07–5.53	0.038	1.30	0.54–3.38	0.567	0.70	0.26–1.94	0.478	0.49	0.17–1.46	0.183	0.56	0.25–1.30	0.172

OR = Odds Ratio; CI = Confidence Interval; *P*: p-value; Erosions: radiographic joint erosions; DAS28-CRP: disease activity score 28-joints with c-reactive protein; Anti-CCP: anti-cyclic citrullinated peptide; IgM-RF: IgM rheumatoid factor; Bio.treatment: biological treatment.

None of the hg clusters were significantly associated with anti-CCP, IgM-RF, DAS28-CRP or biological treatment (Table 4).

When testing the multivariable association between clinico-pathological factors and allocation to biological treatment in Table 5, there were significantly fewer patients from hg cluster JT that received biological treatment (OR = 0.17, 95% CI: 0.03–0.87, p = 0.038). There was a borderline significant association between getting biological treatment and having erosions (OR = 3.61, 95% CI: 1.04–14.45, p = 0.050) and a significant association between biological treatment and having moderate/severe DAS28-CRP (OR = 35.83, 95% CI: 10.18–164.94, p < 0.001). No association was found between biological treatment and anti-CCP or IgM-RF (Table 5).

**Table 5:** Multivariable analysis of associations between clinicopathological factors and allocation to biological treatment.

	Biological treatment		
	OR	(95% CI)	*P*
**Haplogroup cluster:**			
*HV*	1.00		
*UK*	0.52	0.11–2.32	0.385
*JT*	0.17	0.03–0.87	0.038
**Erosion:**			
*Absent*	1.00		
*Present*	3.61	1.04–14.45	0.050
**DAS28-CRP:**			
*Remission/mild*	1.00		
*Moderate/severe*	35.83	10.18–164.94	<0.001
**Anti-CCP:**			
*No*	1.00		
*Yes*	0.70	0.07–4.99	0.740
**IgM-RF:**			
*No*	1.00		
*Yes*	0.35	0.01–3.71	0.439

OR = Odds Ratio; CI = Confidence Interval; *P*: p-value; Erosions: radiographic joint erosions; DAS28-CRP: disease activity score 28-joints with c-reactive protein; Anti-CCP: anti-cyclic citrullinated peptide; IgM-RF: IgM rheumatoid factor.

## 4. Discussion

This study investigated the distribution of mtDNA hgs in patients with RA according to disease activity, presence of rheumatoid factor (and/or anti-CCP antibodies), and biological treatment. The distribution of mtDNA hgs in the RA-cohort was compared with two historical control groups from the background population and the overall distribution of hgs was found to be comparable with the distribution of the background populations. Thus, there does not seem to be an association between the occurrence of RA and mtDNA hgs.

The study demonstrated an association albeit insignificant following Bonferroni’s correction for multiple testing, between radiographic erosions and the hg cluster JT. However, the multivariable analysis showed that significantly fewer patients from the hg cluster JT received biological treatment compared to hg cluster HV. Furthermore, there was a significant association between biological treatment and having moderate/severe DAS28-CRP, but only borderline significance was obtained when radiographic erosions were included in the calculations. The hg cluster JT had a higher frequency of erosions compared to hg cluster HV.

Thus, our results might indicate that patients in hg cluster JT exhibit a higher disease activity. This may be a consequence of the small study sample, as hg H has been associated with increased pathogenicity in degenerative diseases compared to other hgs. A study by Soto-Hermida et al. evaluated the influence of the mtDNA hgs on the progession of knee OA and found cartilage thickness loss to be significantly lower in patients with hgs T [11]. They suggested mtDNA hg T to have a higher capacity to cope with oxidative stress than hg H [11]. This was supported by two other studies indicating a higher vulnerability of hg H to ROS than hg T [22,23]. Previous studies have shown that higher energy turnover of the cell and protonmotive force is positively correlated to ROS production [24,25]. Thereby expecting the HV cluster to associate with a higher amount of erosions.

An imbalance between ROS production and elimination is thought to lead to the oxidative stress in RA patients which contributes to tissue damage and further to the chronicity of the disease [26]. Oxidative stress has therefore been considered to be involved in the onset of RA [15,26–29].

Measurements on oxidative stress have been challenging, but studies have been performed to analyse whether mtDNA hgs differ in ROS production [22,30–32]. A comparison of the findings of these studies is complicated by the variation in the choice of models, methods and design used. The ROS production have been measured in transmitochondrial cybrids using fluorogenic dyes as MitoSOX and H2DCFDA [30–32]. The cybrid cell-lines were not the same in these studies and the flourogenic dyes have been found controversial as a method for measuring mitochondrial ROS production [33,34]. The mitochondrial ROS production is linked to the mitochondrial membrane potential, that is connected to the rate of OXPHOS [35]. The variation that may exist in OPXHOS function in different hgs is coupled to variation in ROS production. The OXPHOS function in hgs have been analysed using respirometry [30,32,36,37], respiratory gas-exchange [23,38,39], enzymatic assays [22], in cybrids [22,30,32,37], humans [23,38,39], and permeabilized muscle cells [36]. Interestingly, hgs UK and H have been studied by a similar method by Gomez-Duran [32], and Larsen [36], in cybrids and permeabilized muscle fibers, reaching opposite conclusions. This indicates variability in OXPHOS function between mtDNA hgs [23,30,32,36]. There is evidence that ROS are involved in cartilage degradation in vitro and in animal OA models. [40].

A study by Mitsunaga et al. found a significant association between severe erosive RA and mitochondrial respiratory complex-related genes, that were major site of ROS generation [15]. They suggested that ROS were involved in cartilage and bone destruction through activation of osteoclast and fibroblast-like synoviocytes [15].

To our knowledge only one study by Coto-Segura et al. has examined the hgs frequencies in patients with arthritis—psoriatic arthritis (PsA) [41]. Comparing the most common hg H with hg J, they found that hg J was significantly less frequent among patients with PsA, hypothesising a protective effect in their study population [41]. Other studies support the protective effects of hg J when regarding the development of OA [7,10,12].

Our findings, that there are not any significant association between European mtDNA neither with occurrence of RA nor with clinical manifestations may reflect the complexity of RA etiology and pathogenesis, as the mtDNA variation most likely involves many other mitochondrial functions. It is probably not sufficient to interpret the results with reference solely to ROS production. Finally, mtDNA only code for approximately 1% of the mitochondrial proteome [42], so population substratification may also confound the results. However, more studies are needed to confirm our findings and more carefully relate them to clinical and biochemically defined subtypes of RA.

## References

[1] Hagen CM, Aidt FH, Hedley PL, Jensen MK, Havndrup O, Kanters JK, et al Mitochondrial Haplogroups Modify the Risk of Developing Hypertrophic Cardiomyopathy in a Danish Population. PLoS One. 2013; 8: e71904 doi: 10.1371/journal.pone.0071904 . 23940792 10.1371/journal.pone.0071904 PMC3734310

[2] Hagen CM, Aidt FH, Havndrup O, Hedley PL, Jensen MK, Kanters JK, et al Private Mitochondrial DNA Variants in Danish Patients with Hypertrophic Cardiomyopathy. PLoS One. 2015; 10: e0124540 doi: 10.1371/journal.pone.0124540 . 25923817 10.1371/journal.pone.0124540 PMC4414448

[3] Hart A, Samuels D, Hulgan T. The Other Genome: A Systematic Review of Studies of Mitochondrial DNA Haplogroups and Outcomes of HIV Infection and Antiretroviral Therapy. AIDS rev. 2008; 42: 157–162. PMC4001077 24322381

[4] Wallace DC. A Mitochondrial Paradigm of Metabolic and Degenerative Diseases, Aging, and Cancer: A Dawn for Evolutionary Medicine. Annu Rev Genet. 2005; 39: 359–407. doi: 10.1146/annurev.genet.39.110304.095751 . 16285865 10.1146/annurev.genet.39.110304.095751 PMC2821041

[5] Herrnstadt C, Howell N. An evolutionary perspective on pathogenic mtDNA mutations: Haplogroup associations of clinical disorders. Mitochondrion. 2004; 4: 791–798. doi: 10.1016/j.mito.2004.07.041 . 16120433 10.1016/j.mito.2004.07.041

[6] Benn M, Schwartz M, Nordestgaard BG, Tybjærg-Hansen A. Mitochondrial haplogroups: Ischemic cardiovascular disease, other diseases, mortality, and longevity in the general population. Circulation. 2008; 117: 2492–2501. doi: 10.1161/CIRCULATIONAHA.107.756809 . 18458168 10.1161/CIRCULATIONAHA.107.756809

[7] Rego-Pérez I, Fernández-Moreno M, Fernández-López C, Arenas J, Blanco FJ. Mitochondrial DNA haplogroups: Role in the prevalence and severity of knee osteoarthritis. Arthritis Rheum. 2008; 58: 2387–2396. doi: 10.1002/art.23659 . 18668590 10.1002/art.23659

[8] van der Walt JM, Nicodemus KK, Martin ER, Scott WK, Nance MA, Watts RL, et al Mitochondrial polymorphisms significantly reduce the risk of Parkinson disease. Am J Hum Genet. 2003; 72: 804–811. doi: 10.1086/373937 . 12618962 10.1086/373937 PMC1180345

[9] van der Walt JM, Dementieva YA, Martin ER, Scott WK, Nicodemus KK, Kroner CC, et al Analysis of European mitochondrial haplogroups with Alzheimer disease risk. Neurosci Lett. 2004; 365: 28–32. doi: 10.1016/j.neulet.2004.04.051 . 15234467 10.1016/j.neulet.2004.04.051

[10] Rego-Perez I, Fernández-Moreno M, Deberg M, Pértega S, Fernández-López C, Oreiro N, et al Mitochondrial DNA haplogroups and serum levels of proteolytic enzymes in patients with osteoarthritis. Ann Rheum Dis. 2011; 70: 646–652. doi: 10.1136/ard.2010.133637 . 21177294 10.1136/ard.2010.133637

[11] Soto-Hermida A, Fernández-Moreno M, Oreiro N, Fernández-López C, Pértega S, Cortés-Pereira E, et al Mitochondrial DNA (mtDNA) Haplogroups Influence the Progression of Knee Osteoarthritis. Data from the Osteoarthritis Initiative (OAI). PLoS One. 2014; 9: e112735 doi: 10.1371/journal.pone.0112735 . 25390621 10.1371/journal.pone.0112735 PMC4229258

[12] Rego I, Fernández-Moreno M, Fernández-López C, Gómez-Reino JJ, González A, Arenas J, et al Role of European mitochondrial DNA haplogroups in the prevalence of hip osteoarthritis in Galicia, Northern Spain. Ann Rheum Dis. 2010; 69: 210–213. doi: 10.1136/ard.2008.105254 . 19224903 10.1136/ard.2008.105254

[13] Hudson G, Gomez-Duran A, Wilson IJ, Chinnery PF. Recent Mitochondrial DNA Mutations Increase the Risk of Developing Common Late-Onset Human Diseases. PLoS Genet. 2014; 10: e1004369 doi: 10.1371/journal.pgen.1004369 . 24852434 10.1371/journal.pgen.1004369 PMC4031051

[14] McInnes IB, Schett G. The pathogenesis of rheumatoid arthritis. N Engl J Med. 2011; 365: 2205–2219. doi: 10.1056/NEJMra1004965 . 22150039 10.1056/NEJMra1004965

[15] Mitsunaga S, Hosomichi K, Okudaira Y, Nakaoka H, Suzuki Y, Kuwana M, et al Aggregation of rare/low-frequency variants of the mitochondria respiratory chain-related proteins in rheumatoid arthritis patients. J Hum Genet. 2015; 60: 449–454. doi: 10.1038/jhg.2015.50 . 26016412 10.1038/jhg.2015.50

[16] Aletaha D, Neogi T, Silman AJ, Funovits J, Felson DT, Bingham CO, et al 2010 Rheumatoid arthritis classification criteria: An American College of Rheumatology/European League Against Rheumatism collaborative initiative. Arthritis Rheum. 2010; 62: 2569–2581. doi: 10.1002/art.27584 . 20872595 10.1002/art.27584

[17] PhyloTree [Internet]. 2014 [cited 2014 May 18]. Available from: www.phylotree.org.

[18] D a n b i o Årsrapport [Internet]. 2011 [cited 2016 June 26]. 1–16. Available from: https://danbio-online.dk/formidling.

[19] Baggrundsnotat for biologisk behandling af Reumatoid Artritis (RA) [Internet]. 2014 [cited 2016 June 26]. 1–13. Available from: http://www.regioner.dk/media/1823/reuma-baggr-med-bilag-januar-2014.pdf.

[20] Mikkelsen M, Sørensen E, Rasmussen EM, Morling N. Mitochondrial DNA HV1 and HV2 variation in Danes. Forensic Sci Int Genet. 2010; 4: 87–88. doi: 10.1016/j.fsigen.2009.07.007 . 20457038 10.1016/j.fsigen.2009.07.007

[21] R Core team. R: A language and environment for statistical computing [Internet]. 2013 [cited 2015 Nov 22]. Available from: http://www.R-project.org/.

[22] Mueller EE, Brunner SM, Mayr JA, Stanger O, Sperl W, Kofler B. Functional Differences between Mitochondrial Haplogroup T and Haplogroup H in HEK293 Cybrid Cells. PLoS One. 2012; 7: e52367 doi: 10.1371/journal.pone.0052367 . 23300652 10.1371/journal.pone.0052367 PMC3530588

[23] Martínez-Redondo D, Marcuello A, Casajús JA, Ara I, Dahmani Y, Montoya J, et al Human mitochondrial haplogroup H: The highest VO2max consumer—Is it a paradox? Mitochondrion. Mitochondria Research Society; 2010; 10: 102–107. doi: 10.1016/j.mito.2009.11.005 . 19900587 10.1016/j.mito.2009.11.005

[24] Skulachev VP. Uncoupling: New approaches to an old problem of bioenergetics. Biochim Biophys Acta—Bioenerg. 1998; 1363: 100–124. . 9507078 10.1016/s0005-2728(97)00091-1

[25] Abele D, Heise K, Pörtner HO, Puntarulo S. Temperature-dependence of mitochondrial function and production of reactive oxygen species in the intertidal mud clam Mya arenaria. J Exp Biol. 2002; 205: 1831–1841. . 12077159 10.1242/jeb.205.13.1831

[26] Mateen S, Moin S, Khan AQ, Zafar A, Fatima N. Increased Reactive Oxygen Species Formation and Oxidative Stress in Rheumatoid Arthritis. PLoS One. 2016; 11: e0152925 doi: 10.1371/journal.pone.0152925 . 27043143 10.1371/journal.pone.0152925 PMC4820274

[27] Veselinovic M, Barudzic N, Vuletic M, Zivkovic V, Tomic-Lucic A, Djuric D, et al Oxidative stress in rheumatoid arthritis patients: Relationship to diseases activity. Mol Cell Biochem. 2014; 391: 225–232. doi: 10.1007/s11010-014-2006-6 . 24610042 10.1007/s11010-014-2006-6

[28] Rall LC, Roubenoff R, Meydani SN, Han SN. (8-OHdG) as a marker of oxidative stress in rheumatoid arthritis and aging : Effect of progressive resistance training. J Nutr Biochem. 2000; 2863: 581–584. 10.1016/s0955-2863(00)00123-6 11137896

[29] Phillips D, Dias H, Kitas G, Griffiths H. Aberrant reactive oxygen and nitrogen species generation in rheumatoid arthritis (RA): Causes and consequences for immune function, cell survival, and therapeutic intervention. Antioxidants Redox Signal. 2010; 12: 743–785. doi: 10.1089/ars.2009.2607 . 19686039 10.1089/ars.2009.2607

[30] Kenney MC, Chwa M, Atilano SR, Falatoonzadeh P, Ramirez C, Malik D, et al Molecular and bioenergetic differences between cells with African versus European inherited mitochondrial DNA haplogroups: Implications for population susceptibility to diseases. Biochim Biophys Acta—Mol Basis Dis. 2014; 1842: 208–219. doi: 10.1016/j.bbadis.2013.10.016 . 24200652 10.1016/j.bbadis.2013.10.016 PMC4326177

[31] Kenney MC, Chwa M, Atilano SR, Pavlis JM, Falatoonzadeh P, Ramirez C, et al Mitochondrial DNA Variants Mediate Energy Production and Expression Levels for CFH, C3 and EFEMP1 Genes: Implications for Age-Related Macular Degeneration. PLoS One. 2013; 8: e54339 doi: 10.1371/journal.pone.0054339 . 23365660 10.1371/journal.pone.0054339 PMC3554762

[32] Gómez-Durán A, Pacheu-Grau D, López-Gallardo E, Díez-Sánchez C, Montoya J, López-Pérez MJ, et al Unmasking the causes of multifactorial disorders: OXPHOS differences between mitochondrial haplogroups. Hum Mol Genet. 2010; 19: 3343–3353. doi: 10.1093/hmg/ddq246 . 20566709 10.1093/hmg/ddq246

[33] Kalyanaraman B, Darley-Usmar V, Davies KJA, Dennery PA, Forman HJ, Grisham MB, et al Measuring reactive oxygen and nitrogen species with fluorescent probes: Challenges and limitations. Free Radic Biol Med. 2012; 52: 1–6. doi: 10.1016/j.freeradbiomed.2011.09.030 . 22027063 10.1016/j.freeradbiomed.2011.09.030 PMC3911769

[34] Zielonka J, Kalyanaraman B. “ROS-generating mitochondrial DNA mutations can regulate tumor cell metastasis”-a critical commentary. Free Radic Biol Med. 2008; 45: 1217–1219. doi: 10.1016/j.freeradbiomed.2008.07.025 . 18789385 10.1016/j.freeradbiomed.2008.07.025 PMC3595710

[35] Chen Y-R, Zweier JL. Cardiac mitochondria and reactive oxygen species generation. Circ Res. 2014; 114: 524–537. doi: 10.1161/CIRCRESAHA.114.300559 . 24481843 10.1161/CIRCRESAHA.114.300559 PMC4118662

[36] Larsen S, Díez-Sánchez C, Rabøl R, Ara I, Dela F, Helge JW. Increased intrinsic mitochondrial function in humans with mitochondrial haplogroup H. Biochim Biophys Acta—Bioenerg. 2014; 1837: 226–231. doi: 10.1016/j.bbabio.2013.10.009 . 24184346 10.1016/j.bbabio.2013.10.009

[37] Amo T, Brand MD. Were inefficient mitochondrial haplogroups selected during migrations of modern humans? A test using modular kinetic analysis of coupling in mitochondria from cybrid cell lines. Biochem J. 2007; 404: 345–351. doi: 10.1042/BJ20061609 . 17355224 10.1042/BJ20061609 PMC1868799

[38] Marcuello A, Martínez-Redondo D, Dahmani Y, Casajús JA, Ruiz-Pesini E, Montoya J, et al Human mitochondrial variants influence on oxygen consumption. Mitochondrion. 2009; 9: 27–30. doi: 10.1016/j.mito.2008.10.002 . 18952007 10.1016/j.mito.2008.10.002

[39] Marcuello A, Martínez-Redondo D, Dahmani Y, Terreros JL, Aragonés T, Casajús JA, et al Steady exercise removes VO2max difference between mitochondrial genomic variants. Mitochondrion. 2009; 9: 326–330. doi: 10.1016/j.mito.2009.04.007 . 19427920 10.1016/j.mito.2009.04.007

[40] Henrotin Y, Kurz B. Antioxidant to treat osteoarthritis: Dream or reality? Curr Drug Targets. 2007; 8: 347–357. . 17305512 10.2174/138945007779940151

[41] Coto-Segura P, Santos-Juanes J, Gómez J, Alvarez V, Díaz M, Alonso B, et al Common European Mitochondrial Haplogroups in the Risk for Psoriasis and Psoriatic Arthritis. Genet Test Mol Biomarkers. 2012; 16: 621–623. doi: 10.1089/gtmb.2011.0266 . 22191676 10.1089/gtmb.2011.0266

[42] Pagliarini DJ, Calvo SE, Chang B, Sheth SA, Vafai SB, Ong SE, et al A Mitochondrial Protein Compendium Elucidates Complex I Disease Biology. Cell. 2008; 134: 112–123. doi: 10.1016/j.cell.2008.06.016 . 18614015 10.1016/j.cell.2008.06.016 PMC2778844

